# Outcome of Patients With Both Moderate Aortic Stenosis and Moderate Mitral Stenosis

**DOI:** 10.1016/j.shj.2023.100183

**Published:** 2023-04-25

**Authors:** Takafumi Yamane, Ken Kuwajima, Shunsuke Kagawa, Hiroko Hasegawa, Florian Rader, Robert J. Siegel, Takahiro Shiota

**Affiliations:** Smidt Heart Institute, Cedars-Sinai Medical Center, Los Angeles, California, USA

**Keywords:** Aortic stenosis, Heart failure, Mitral stenosis, Stroke volume index

## Abstract

**Aims:**

This study aimed to investigate the symptoms and prognosis of patients with both moderate aortic stenosis (AS) and mitral stenosis (MS).

**Methods and Results:**

We studied 82 patients with moderate AS and MS diagnosed via transthoracic echocardiography. The patients had a mean age of 79 ± 13 years and 95% of patients had degenerative MS. Out of 82 patients, 34 (41%) had heart failure (HF) symptoms (New York Heart Association class ≥ Ⅱ) or a history of HF admission. Left ventricular ejection fraction, stroke volume index, atrial fibrillation, and right ventricular systolic pressure were independent determinants of HF symptoms. The median follow-up duration was 3.2 (interquartile range, 1.0-4.9) years and clinical events occurred in 48 (59%) patients, including death in 11 (13%) patients, aortic or mitral valve interventions in 22 (27%) patients, and HF hospitalization in 15 (18%) patients. The 5-year survival free of the combined endpoint of aortic or mitral valve interventions, HF hospitalization, or death was 19%. A multivariate predictor of clinical events was HF symptoms (hazard ratio [HR], 2.32; 95% confidence interval [CI], 1.30-4.14; *p* = 0.0045). Kaplan-Meier survival at 5 years was 61% without intervention and HF symptoms were not associated with mortality.

**Conclusions:**

Among patients with both moderate AS and MS, left ventricular ejection fraction, stroke volume index, atrial fibrillation, and right ventricular systolic pressure were strong determinants of HF symptoms. HF symptoms were independently predictive of clinical events.

## Highlights

• Degenerative causes of aortic and mitral stenosis have become more common.
• Among patients with both moderate aortic and mitral stenosis, 41% were symptomatic.
• Stroke volume index was associated with heart failure symptoms.
• The 5-year survival free of the combined endpoint was 19%.
• Heart failure symptoms were associated with the combined endpoint but not mortality.

## Introduction

In previous decades, rheumatic disease used to be the most frequent etiology of valvular heart disease. However, in industrialized countries, the incidence of rheumatic heart disease has substantially decreased.^1^ On the other hand, degenerative causes of valvular heart diseases, especially aortic stenosis (AS), have become more common in recent years.^2^ Degenerative AS is characterized by calcification of the aortic cusps.^3^^,^^4^ Alongside AS, degenerative causes of mitral stenosis (MS), mainly due to mitral annular calcification, have become more prevalent.^5^^,^^6^ As a result, degenerative AS and mitral annular calcification resulting in significant MS may coexist.^7^ In fact, the prevalence of significant MS is reported to be as much as 12% in patients undergoing surgical and transcatheter aortic valve replacement.^8^^,^^9^ Patients who have both AS and MS may develop symptoms even if the individual stenotic lesions are not severe. Such patients with combined multiple valve diseases have poor prognosis once they develop symptoms.^10^ However, to the best of our knowledge, there is a lack of data regarding patients with moderate AS and moderate MS, and their treatment is not addressed by the guidelines.^11^ The factors associated with heart failure (HF) symptoms and prognosis in these patients remain unknown. Therefore, this study aimed to 1) investigate factors related to the symptoms of patients who have both moderate AS and moderate MS; and 2) identify the predictors of clinical events in patients who have both moderate AS and moderate MS.

## Methods

### Participants

**Figure 1:**
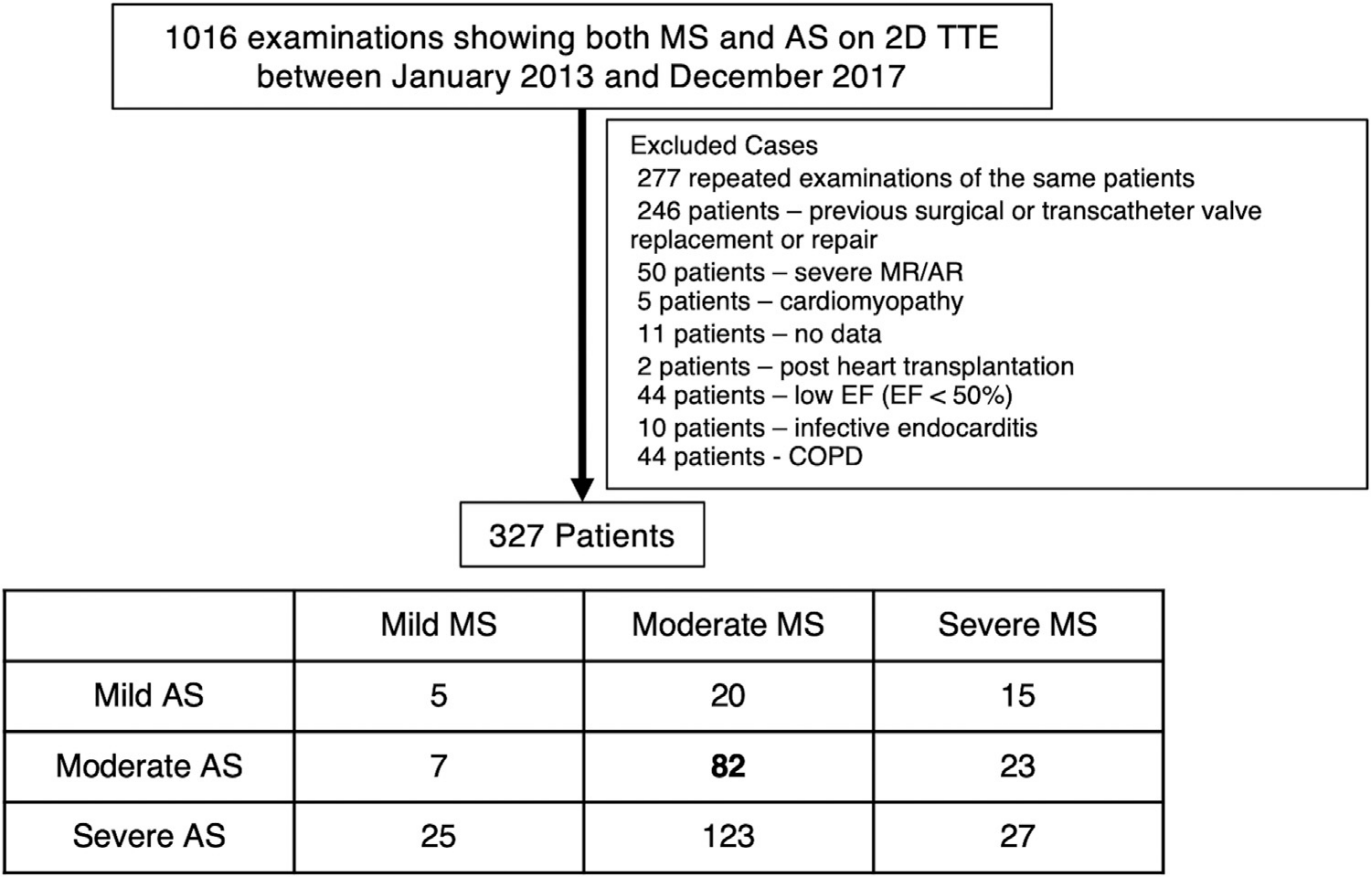
**Flowchart of the study population**. Abbreviations: 2D, two-dimensional; AR, aortic regurgitation; AS, aortic stenosis; COPD, chronic obstructive pulmonary disease; MR, mitral regurgitation; MS, mitral stenosis; SVI, stroke volume index, TTE, transthoracic echocardiography.

We retrospectively reviewed 1016 consecutive patients diagnosed with AS and MS via two-dimensional transthoracic echocardiography (TTE) at our heart institute between January 2013 and December 2017. We excluded repeat examinations of the same patients. Additionally, the following exclusion criteria were applied: age <18 years, previous surgical or transcatheter valve replacement or valve repair, reduced left ventricular (LV) systolic function (LV ejection fraction [LVEF] < 50%), moderate or greater mitral regurgitation, moderate or greater aortic regurgitation, cardiomyopathy, endocarditis, chronic obstructive pulmonary disease, prior heart transplantation, and insufficient medical record data. Among the remaining 327 patients, 82 patients had both moderate AS and MS as shown in Figure 1. Moderate AS was defined as either 1) aortic peak jet velocity of >2.5m/s and less than 4 m/s; 2) mean transaortic pressure gradient of 20 to 40 mmHg; or 3) aortic peak jet velocity of <2.5 m/s, mean transaortic pressure gradient of <20 mmHg and aortic valve area of 1.0 to 1.5 cm^2^. Moderate MS was defined as mean transmitral pressure gradient of 5 to 10 mmHg. This retrospective observational study was approved by the institutional ethical board, with a waiver of individual informed consent due to the retrospective nature of the study.

### Clinical Characteristics

All data were collected from the medical charts and our echocardiographic database. Baseline patient characteristics and laboratory data were collected on the same date as TTE, or when not possible, within 3 days prior to the TTE. The symptoms of each patient were evaluated via the New York Heart Association class, which was determined by the attending physician or a senior cardiologist. Patients were considered to be symptomatic when they had HF symptoms (New York Heart Association class ≥ Ⅱ) or had a history of HF admission. Patients with end-stage renal disease were defined as those undergoing hemodialysis or those who received kidney transplants. Patients with atrial fibrillation were defined as those with permanent, persistent, or paroxysmal atrial fibrillation. Inactivity was defined as requirement for assistance with activities of daily living and self-care.^12^

### Echocardiographic Data

Comprehensive TTE was performed according to the guidelines set by the American Society of Echocardiography.^13^ The iE33 ultrasound system (Philips Medical Systems, Andover, MA) equipped with an S5-1 phased array transducer was used. In patients with atrial fibrillation, all measurements were an average of at least 5 cardiac cycles. LVEF and ventricular volumes were calculated using the biplane Simpson’s method. LV stroke volume was derived using the time-velocity integral of LV outflow tract, assuming a circular geometry of the LV outflow tract. The stroke volume index (SVI) was calculated as stroke volume divided by body surface area. Aortic valve area was derived from the continuity equation.^14^ Mitral valve area was calculated by either pressure half-time or planimetry. The severities of mitral regurgitation, aortic regurgitation, and tricuspid regurgitation were assessed using an integrated approach as recommended by current guidelines.^15^ Right ventricular systolic pressure (RVSP) was calculated from right atrial pressure and the systolic pressure gradient between right ventricular and right atrium calculated by applying the modified Bernoulli equation.^16^ Right atrial pressure was estimated by the diameter of inferior vena cava and its response to a sniff and was classified using 3 grades: 3, 8, and 15 mm Hg.

### Follow-Up and Clinical Outcomes

The primary clinical outcome of interest in the study was a composite of all-cause mortality, hospitalization for HF, and aortic or mitral valve intervention. Hospitalization for HF was defined as a hospital admission with HF as the primary diagnosis. Another clinical outcome was all-cause mortality, censoring patients at aortic or mitral valve interventions. Censoring was defined as the day of procedure in patients undergoing aortic or mitral valve intervention. The decision to perform aortic or mitral valve intervention was at the discretion of the patients’ treating cardiologists and cardiovascular surgeons. The follow-up information was obtained by a detailed review of all medical records.

### Statistical Analysis

Categorical variables are shown as numbers and percentages and were compared using the chi-squared test or Fisher’s exact test as appropriate. Continuous variables are expressed as mean and standard deviation or as median and interquartile range (IQR). Based on their distribution, qualitatively judged via histogram and Q-Q plot, continuous variables were compared using Student’s *t*-test or Wilcoxon rank-sum test as appropriate. We used univariable logistic regression analysis to identify variables that were significantly related to the presence of HF symptoms, and all variables with suspected clinical relevance were entered. Covariates with statistical significance (*p* < 0.05) at univariate logistic regression were included in the multivariate logistic regression to identify independently associated parameters with the presence of HF symptoms. Regarding the parameters of AS and MS severity, we applied mean pressure gradient for univariate analysis. The estimated risk of a given variable was expressed via an odds ratio (OR) with its corresponding 95% confidence intervals (CIs). Receiver operating characteristic curves were generated to determine the optimal cutoff value of SVI for predicting the presence of HF symptoms. Kaplan-Meier estimates were used to calculate the probability of event-free survival. Cox proportional hazards regression analysis was used to examine the effect of the clinical and echocardiographic variables on the clinical endpoints. Cox proportional hazard models included age, sex, and significant variables (*p* < 0.05) in the univariate Cox analysis, and hazard ratios (HRs) and 95% CI were calculated. We also conducted sensitivity analysis, analyzing after excluding patients with aortic valve area less than 1.0 cm^2^.

Statistical analyses were performed using the statistical software program JMP 16.1.0 (SAS Institute Inc., Cary, North Carolina, USA), and *p* < 0.05 was considered statistically significant.

## Results

### Patient Clinical and Echocardiographic Characteristics

The mean patient age was 79 ± 13 years (IQR: 70-89), and 70% of patients were female. The etiology of MS was degenerative in 95% of patients. The clinical characteristics of 82 patients with both moderate MS and AS are shown in Table 1. At the time of index TTE, 34 patients (41%) had HF symptoms, whereas 48 patients (59%) were asymptomatic. Symptomatic patients had a higher prevalence of atrial fibrillation than asymptomatic patients. Echocardiographic characteristics of patients are shown in Table 2. Symptomatic patients had a lower LVEF (66 ± 8% vs. 70 ± 7%, *p* = 0.015) and SVI (43.7 ± 15.7 vs. 53.2 ± 18.3 ml/m^2^, *p* = 0.016) than asymptomatic patients. Moreover, symptomatic patients were more likely to have rheumatic valvular heart disease, a larger left atrial volume index and right atrial area, and higher RVSP than asymptomatic patients.

**Table 1:** Baseline demographic and clinical characteristics of patients stratified according to heart failure symptoms

	All patients (n = 82)	Asymptomatic (n = 48)	Symptomatic (n = 34)	*p* value
Age, y	79 ± 13	80 ± 12	77 ± 13	0.28
Female patients	57 (70)	32 (67)	25 (74)	0.50
Body mass index	26.7 ± 6.9	26.7 ± 6.9	26.7 ± 7.0	0.99
Body surface area	1.76 ± 0.29	1.75 ± 0.27	1.78 ± 0.31	0.63
Heart rate, bpm	75 ± 12	76 ± 10	74 ± 14	0.57
Systolic BP, mm Hg	132 ± 18	132 ± 16	132 ± 20	0.86
Diastolic BP, mm Hg	65 ± 13	64 ± 14	65 ± 12	0.76
Hypertension	70 (85)	38 (79)	32 (94)	0.059
Dyslipidemia	55 (67)	29 (60)	26 (76)	0.12
Diabetes	31 (38)	18 (38)	13 (38)	0.95
Smoking history	28 (34)	16 (33)	12 (35)	0.85
End-stage renal disease	17 (21)	9 (19)	8 (24)	0.60
Cardiac implantable electronic device	7 (9)	5 (10)	2 (6)	0.46
Atrial fibrillation	16 (20)	5 (10)	11 (32)	0.014
Coronary artery disease	22 (27)	12 (25)	10 (29)	0.66
Inactivity	16 (20)	11 (23)	5 (15)	0.35
Laboratory data				
BNP, pg/mL	462 [131-523]	411 [106-490]	534 [170-609]	0.30
Creatinine, mg/dL	1.9 [0.8-1.4]	1.7 [0.8-1.5]	2.1 [0.9-1.4]	0.44
Hemoglobin, g/dL	10.7 ± 1.7	10.8 ± 1.7	10.5 ± 1.7	0.46
Total bilirubin, mg/dL	0.8 [0.4-0.9]	0.7 [0.4-0.8]	0.9 [0.4-1.0]	0.31
Albumin, g/dL	3.7 ± 0.6	3.7 ± 0.6	3.7 ± 0.6	0.80
Medication				
Beta blocker	37 (45)	18 (38)	19 (56)	0.099
ACEI or ARB	45 (55)	24 (50)	21 (62)	0.29
Loop diuretics	30 (37)	15 (31)	15 (44)	0.23
Statin	45 (55)	25 (52)	20 (59)	0.55

*Notes*. Continuous variables are presented as mean ± standard deviation unless otherwise noted as median [interquartile range]; categorical variables are summarized as n (%).

ACEI, angiotensin-converting enzyme inhibitor; ARB, angiotensin receptor blocker; BNP, brain natriuretic peptide; BP, blood pressure.

**Table 2:** Baseline echocardiographic characteristics of patients stratified according to heart failure symptoms

	All patients (n = 82)	Asymptomatic (n = 48)	Symptomatic (n = 34)	*p* value
LV end-diastolic volume, mL	65 ± 33	64 ± 36	66 ± 29	0.71
LV end-systolic volume, mL	21 ± 13	20 ± 14	23 ± 13	0.33
Interventricular septum thickness, cm	1.29 ± 0.26	1.25 ± 0.27	1.34 ± 0.23	0.093
LV posterior wall thickness, cm	1.22 ± 0.22	1.21 ± 0.22	1.22 ± 0.23	0.95
LV ejection fraction, %	68 ± 8	70 ± 7	66 ± 8	0.015
Stroke volume index, mL/m^2^	49.3 ± 17.8	53.2 ± 18.3	43.7 ± 15.7	0.016
Aortic valve				
Peak aortic velocity, m/s	3.3 ± 0.5	3.2 ± 0.5	3.3 ± 0.5	0.84
Peak pressure gradient, mm Hg	45 ± 12	45 ± 13	45 ± 12	0.84
Mean pressure gradient, mm Hg	25 ± 7	25 ± 6	25 ± 7	0.98
Aortic valve area, cm^2^	1.18 ± 0.33	1.22 ± 0.28	1.14 ± 0.39	0.32
Mitral valve				
MAC	76 (93)	46 (96)	30 (88)	0.20
Rheumatic mitral stenosis	4 (5)	0 (0)	4 (12)	0.015
Mean pressure gradient, mm Hg	6.5 ± 1.5	6.4 ± 1.6	6.6 ± 1.4	0.38
Peak pressure gradient, mm Hg	16.0 ± 4.4	15.1 ± 4.2	17.3 ± 4.4	0.023
Left atrial volume index, mL/m^2^	46.3 ± 17.2	43.1 ± 18.2	50.8 ± 14.7	0.048
Right atrial area, cm^2^	15.6 ± 5.9	15.7 ± 4.7	20.2 ± 6.3	0.0006
RV end-diastolic diameter, cm	3.5 ± 0.8	3.5 ± 0.8	3.6 ± 0.8	0.33
Tricuspid regurgitation ≥ moderate	9 (11)	4 (8)	5 (15)	0.37
RV systolic pressure, mm Hg	41 ± 13	38 ± 10	46 ± 16	0.0058
Estimated right atrial pressure >15 mm Hg	8 (10)	3 (6)	5 (15)	0.21

*Notes*. Continuous variables are presented as mean ± standard deviation unless; categorical variables are summarized as n (%).

LV, left ventricular; MAC, mitral annular calcification; RV, right ventricular.

### Determinants of HF Symptoms

**Figure 2:**
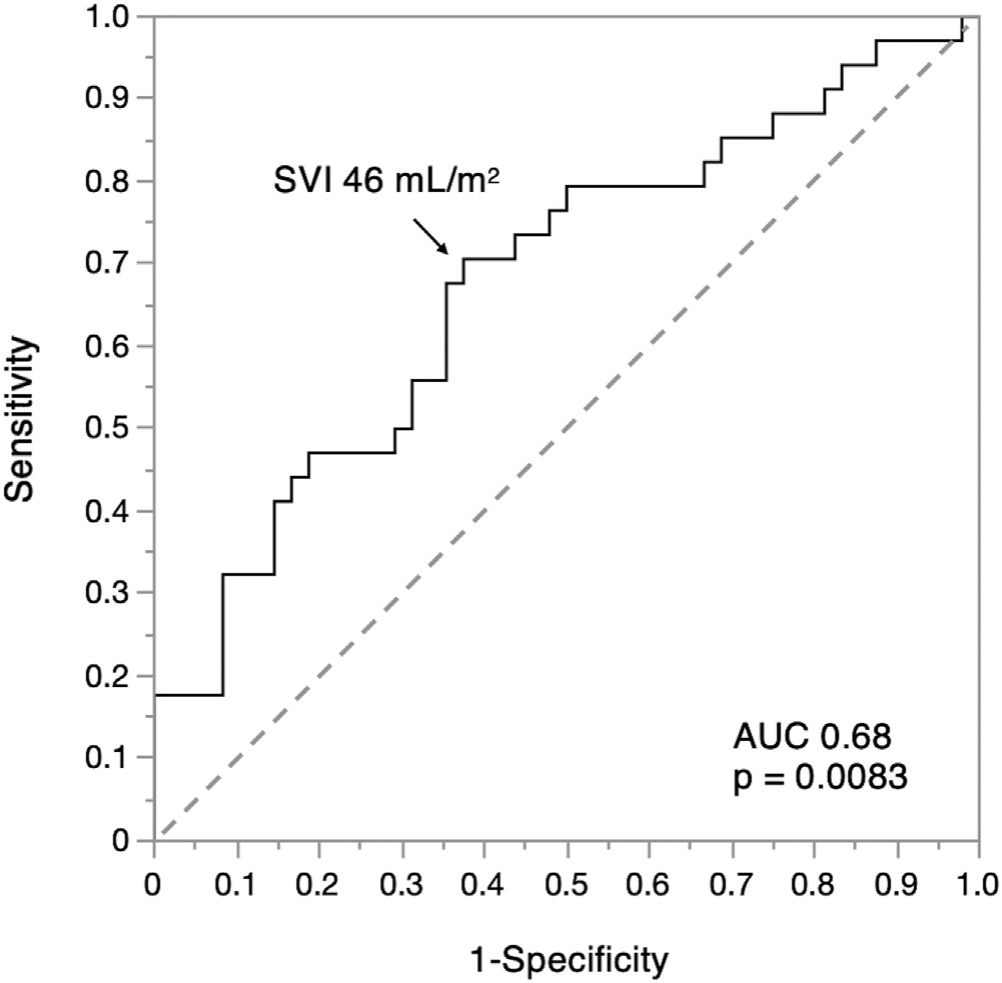
**Receiver operating curve for determining the optimal cutoff value of SVI for HF symptoms in patients with both moderate AS and MS.** The optimal cutoff value of SVI was 46 mL/m^2^ with a sensitivity of 71% and specificity of 63%. The area under the curve was 0.68 (*p* = 0.0083). Abbreviations: AUC, area under the curve; AS, aortic stenosis; HF, heart failure; MS, mitral stenosis; SVI, stroke volume index.

The results of univariate and multivariate analyses for the determinants of HF symptoms in patients with both moderate AS and MS are shown in Table 3. Atrial fibrillation (OR, 5.52; 95% CI, 1.15-25.7; *p* = 0.032), LVEF (OR, 0.91; 95% CI, 0.83-0.98; *p* = 0.011), SVI (OR, 0.95; 95% CI, 0.90-0.99; *p* = 0.021), and RVSP (OR, 1.05; 95% CI, 1.00-1.11; *p* = 0.042) were independent factors associating with HF symptoms. Afterward, a receiver operator characteristic curve was generated to determine the optimal cutoff value for SVI for HF symptoms. The optimal cutoff value of SVI was 46 mL/m^2^ with a sensitivity of 71% and specificity of 63%. The area under the curve was 0.68 (*p* = 0.0083) (Figure 2). On sensitivity analysis, excluding 14 patients with aortic valve area <1.0 cm2, LVEF, SVI, and RVSP were independent factors associating with HF symptoms in agreement with the results for the whole group (Supplemental Table 1).

**Table 3:** Univariate and multivariate analysis determining heart failure symptoms in patients with moderate aortic and mitral stenosis

	Univariate analysis			Multivariate analysis		
	OR	95% CI	*p* value	OR	95% CI	*p* value
Age, y	0.98	0.95-1.02	0.28			
Male	0.72	0.27-1.88	0.50			
Hypertension	4.21	1.02-28.7	0.047	3.04	0.62-22.7	0.18
Diabetes mellitus	1.03	0.41-2.55	0.95			
End-stage renal disease	1.33	0.45-3.93	0.60			
Atrial fibrillation	4.11	1.33-14.4	0.014	5.52	1.15-25.7	0.032
Coronary artery disease	1.25	0.46-3.36	0.66			
Systolic BP, mm Hg	1.00	0.98-1.03	0.86			
Hemoglobin, g/dL	0.91	0.69-1.18	0.46			
BNP, pg/mL	1.00	1.00-1.00	0.29			
Inactivity	0.58	0.17-1.79	0.35			
LV end-diastolic volume, mL	1.00	0.99-1.02	0.71			
LV ejection fraction, %	0.93	0.87-0.99	0.014	0.91	0.83-0.98	0.011
Stroke volume index, mL/m^2^	0.96	0.92-0.99	0.0083	0.95	0.90-0.99	0.021
Aortic valve mean PG, mm Hg	1.00	0.94-1.07	0.98			
Mitral valve mean PG, mm Hg	1.14	0.85-1.53	0.37			
Left atrial volume index, mL/m^2^	1.03	1.00-1.06	0.045	1.02	0.98-1.06	0.32
Right ventricular systolic pressure, mm Hg	1.05	1.01-1.10	0.0055	1.05	1.00-1.11	0.042

BNP, brain natriuretic peptide; BP, blood pressure; CI, confidence interval; LV, left ventricular; OR, odds ratio; PG, pressure gradient.

### Follow-Up and Outcomes

**Figure 3:**
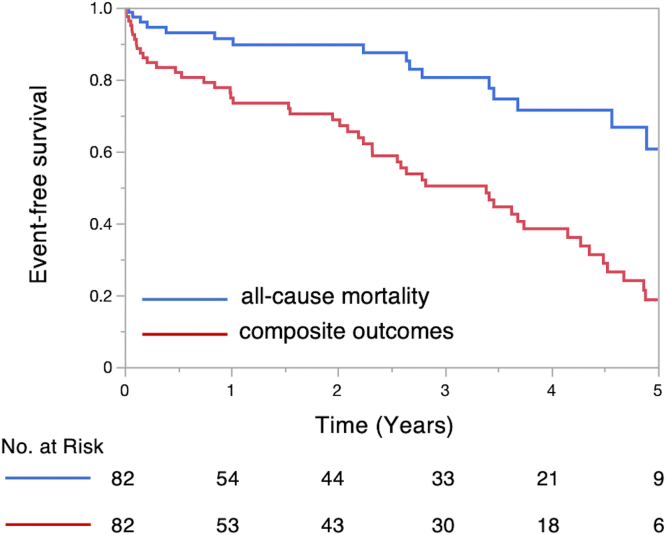
**Kaplan-Meier survival curves for all-cause mortality, and the composite of all-cause mortality, heart failure hospitalization, and aortic or mitral valve intervention.** Event-free survival of all-cause mortality, and the composite of all-cause mortality, heart failure, hospitalization and aortic or mitral valve intervention.

The median follow-up duration was 3.2 years (IQR, 1.0-4.9 years). Of 34 symptomatic patients, 18 patients (53%) underwent aortic or mitral valve interventions. Of 48 patients who were asymptomatic at index TTE, 10 patients (21%) underwent aortic or mitral valve interventions. Among 28 patients receiving aortic or mitral valve interventions, all patients underwent aortic valve replacement (5 surgical, 23 transcatheter) and 6 patients underwent mitral valve intervention (5 surgical, 1 transcatheter). During the follow-up period, events occurred in 48 (59%) patients, including death in 11 (13%) patients, aortic or mitral valve interventions in 22 (27%) patients, and HF hospitalization in 15 (18%) patients. The survival free of the combined endpoint of aortic or mitral interventions, HF hospitalization, and death was 75% at 1 year, 50% at 3 years, and 19% at 5 years, as shown in Figure 3. HF symptoms at index TTE (HR, 2.32; 95% CI, 1.30-4.14; *p* = 0.0045) were independently associated with the combined endpoint (Table 4). On sensitivity analysis, HF symptoms at index TTE were independently associated with the combined endpoint (Supplemental Table 2).

**Table 4:** Univariate and multivariate analysis for predictors of the composite of all-cause mortality, HF hospitalization, and aortic or mitral valve interventions in patients with moderate aortic and mitral stenosis

	Univariate analysis			Multivariate analysis		
	HR	95% CI	*p* value	HR	95% CI	*p* value
Age, y	0.98	0.96-1.01	0.17	0.99	0.97-1.01	0.37
Male	1.39	0.76-2.55	0.28	1.34	0.72-2.52	0.36
Hypertension	1.22	0.52-2.87	0.65			
Diabetes mellitus	1.10	0.62-1.95	0.75			
End-stage renal disease	1.45	0.74-2.81	0.28			
Atrial fibrillation	1.81	0.91-3.57	0.090			
Coronary artery disease	1.08	0.57-2.04	0.81			
HF symptoms	2.32	1.31-4.12	0.0041	2.32	1.30-4.14	0.0045
Hemoglobin, g/dL	0.86	0.72-1.03	0.088			
BNP, pg/mL	1.00	1.00-1.00	0.49			
Inactivity	1.63	0.77-3.45	0.20			
LV end-diastolic volume, mL	1.00	1.00-1.01	0.24			
LV ejection fraction, %	1.01	0.98-1.05	0.54			
Stroke volume index, mL/m^2^	1.00	0.98-1.01	0.69			
Aortic valve mean PG, mm Hg	1.03	0.99-1.08	0.14			
Mitral valve mean PG, mm Hg	1.06	0.84-1.31	0.59			
Left atrial volume index, mL/m^2^	1.01	0.99-1.03	0.17			
Right ventricular systolic pressure, mm Hg	1.02	0.99-1.04	0.15			

BNP, brain natriuretic peptide; BP, blood pressure; CI, confidence interval; HF, heart failure; HR, hazard ratio; LV, left ventricular; PG, pressure gradient.

Among patients with both moderate AS and moderate MS, the probabilities of survival without intervention were 91% at 1 year, 81% at 3 years, and 61% at 5 years, as shown in Figure 3. Baseline hemoglobin levels (HR, 0.72; 95% CI, 0.52-0.98; *p* = 0.040) and inactivity (HR, 5.71; 95% CI, 1.56-20.9; *p* = 0.0085) were independently associated with a higher all-cause mortality (Table 5). On sensitivity analysis, only hemoglobin levels were independently associated with a higher all-cause mortality (Supplemental Table 3).

**Table 5:** Univariate and multivariate analysis for predictors of all-cause mortality in patients with moderate aortic and mitral stenosis

	Univariate analysis			Multivariate analysis		
	HR	95% CI	*p* value	HR	95% CI	*p* value
Age, y	1.01	0.97-1.06	0.77	1.01	0.95-1.07	0.81
Male	1.73	0.63-4.73	0.28	3.23	0.87-11.9	0.080
Hypertension	0.69	0.20-2.45	0.57			
Diabetes mellitus	1.77	0.68-4.61	0.24			
End-stage renal disease	1.43	0.45-4.54	0.54			
Atrial fibrillation	1.53	0.43-5.45	0.52			
Coronary artery disease	0.89	0.29-2.75	0.84			
HF symptoms	0.74	0.26-2.09	0.57			
Hemoglobin, g/dL	0.67	0.49-0.91	0.012	0.72	0.52-0.98	0.040
BNP, pg/mL	1.00	1.00-1.00	0.51			
Inactivity	3.97	1.49-10.6	0.0058	5.71	1.56-20.9	0.0085
LV end-diastolic volume, mL	1.01	0.99-1.02	0.20			
LV ejection fraction, %	1.03	0.97-1.09	0.36			
Stroke volume index, mL/m^2^	1.01	0.99-1.03	0.19			
Aortic valve mean PG, mm Hg	1.02	0.94-1.09	0.63			
Mitral valve mean PG, mm Hg	1.02	0.66-1.47	0.93			
Left atrial volume index, mL/m^2^	1.01	0.98-1.03	0.57			
Right ventricular systolic pressure, mm Hg	1.01	0.97-1.05	0.53			

BNP, brain natriuretic peptide; BP, blood pressure; CI, confidence interval; HF, heart failure; HR, hazard ratio; LV, left ventricular; PG, pressure gradient.

## Discussion

The main findings of the present study were as follows: 1) among 82 patients with both moderate AS and MS, 34 patients (41%) had HF symptoms; 2) atrial fibrillation, LVEF, SVI, and pulmonary hypertension were independently associated with HF symptoms in patients with moderate AS and MS; 3) the 5-year survival free of the combined endpoint of valve intervention, HF hospitalization, or all-cause mortality was 19% at 5 years, and HF symptoms were independently associated with the combined endpoint; and 4) the 5-year overall survival without intervention was 61%, and baseline hemoglobin levels and inactivity were independently associated with all-cause mortality.

### Patients With Both Moderate AS and MS

Although there are a few studies about the natural history of patients with both aortic and mitral disease, these studies reporting a mortality of 40% to 50% over a period of 10 or 20 years are from an era when rheumatic heart disease was more prevalent.^17^, ^18^, ^19^ In industrialized countries, the incidence of rheumatic disease has substantially decreased, whereas degenerative causes of valve disease have become more common due to longer life expectancies.^3^^,^^4^^,^^11^ According to Fischer et al.,^20^ concomitant MS was documented in 157 (7.4%) of 2113 patients from a registry in Canada and France undergoing transcatheter aortic valve replacement, with 88% of 157 patients having degenerative MS. In our study of patients with both moderate AS and MS, 95% of patients had degenerative MS. These results suggest that in the current era of interventions, degenerative causes of MS have become more prevalent, but studies of patients with both moderate AS and MS are scarce. Therefore, according to current guidelines, there is no clear consensus regarding the therapeutic strategy for patients with both moderate AS and moderate MS, specifically for early intervention vs. watchful waiting until a severe grade is reached.

Once patients with significant AS develop symptoms, their prognosis dramatically worsens; increased mortality rates are reported among patients before surgical intervention.^21^^,^^22^ Therefore, symptoms can be a clinical marker indicating the timing for intervention.^23^ However, it is often difficult to differentiate dyspnea associated with HF from deconditioning, fatigue, or frailty, especially in elderly patients. In our study, RVSP, a surrogate of pulmonary hypertension, was an independent determinant of HF symptoms. Pulmonary hypertension is a common complication of left-sided heart diseases, including valvular heart diseases, and this frequently occurs as a symptom of the underlying condition.^24^ Our findings that symptomatic patients had significantly higher RVSP suggest that patients’ symptoms were related to HF.

### LVEF and SVI in Patients With Moderate AS and MS

To the best of our knowledge, no previous study has discussed LVEF and SVI in patients with both moderate AS and MS. LVEF used to be considered as preserved when it is ≥50%, and in asymptomatic patients with severe AS, guidelines recommended an LVEF of <50% as the threshold for referral for aortic valve replacement.^25^^,^^26^ However, several reports suggest that the threshold of LVEF <50% for interventions may be too low and even LVEF between 50% and 60% can be concerning.^27^^,^^28^ Accordingly, the updated guidelines now recommended that in asymptomatic patients with severe high gradient AS, aortic valve replacement may be considered when LVEF declines to <60% (Class 2b, evidence level B).^11^ It has also been reported that reduced stroke volume is associated with poor outcomes in patients with severe AS and preserved LVEF.^29^^,^^30^ Stroke volume is influenced by not only valvular obstruction but also LV volume, contractility, filling pressures, and afterload. AS results in LV concentric remodeling, a smaller LV cavity size, and reductions in LV compliance and filling. Furthermore, MS reduces LV preload, while AS increases LV afterload. Consequently, SVI could be negatively affected by both AS and MS. Reportedly, patients with reduced stroke volume had relatively lower LVEF within the normal range.^29^ In our study of patients with both moderate AS and moderate MS, both LVEF and SVI were associated with HF symptoms. Similar to patients with severe AS, these results may reflect that patients with both moderate AS and moderate MS require higher LVEF to preserve stroke volume.

### Prognosis of Patients With Moderate AS and MS

In the present study, the probability of survival without intervention was 61% at 5 years, and similar to HF studies, anemia and inactivity were linked to higher mortality.^31^^,^^32^ Symptoms and echo parameters were not associated with mortality, while symptoms were related to the combined endpoint. Quite a few symptomatic and initially asymptomatic patients with moderate AS and MS underwent interventions, mainly with transcatheter and surgical aortic valve replacement in our study. Successful transcatheter procedures may have affected or improved their symptoms and outcome. In general populations, there are few transcatheter procedures for those with moderate AS and MS. Thus, our results on prognosis cannot apply to general populations. Further research is needed to investigate whether aortic or mitral valve intervention will improve the prognosis of patients with both moderate AS and moderate MS.

### Limitations

The present study had several limitations. First, this was a single-center retrospective study, and we only studied patients who underwent echocardiography, possibly causing selection bias. Second, the assessment of HF symptoms remains challenging. The presence of symptoms is difficult to assess and interpret in elderly patients and retrospective ascertainment is difficult. Third, we did not have data regarding stress tests or hemodynamic catheterization, and thus patients with low-flow, low-gradient severe stenosis may have been included in our study. Fourth, correct assessment of SVI by Doppler is challenging. The elliptical shape of the LV outflow tract and the site for LV outflow tract diameter measurement in patients with calcific AS affect SVI calculation and subsequently may impact our results.

## Conclusion

The 5-year survival free of the combined endpoint of patients with both moderate AS and MS was 19% at 5 years and HF symptoms were associated with poor outcomes. As much as 41% of patients had HF symptoms, and LVEF, SVI, atrial fibrillation, and pulmonary hypertension were strongly associated with HF symptoms in these patients. Therefore, LVEF, SVI, and pulmonary hypertension evaluated via TTE may be clinically useful for evaluating patients with both moderate AS and MS.

## Ethics Statement

The research has adhered to the relevant ethical guidelines at Cedars-Sinai Medical Center.

## Funding

This research did not receive any specific grant from funding agencies in the public, commercial, or not-for-profit sectors.

## Data Availability Statement

The data underlying this article are available in the article and in its online supplementary material.

## Disclosure Statement

Dr. Rader is a consultant at MyoKardia, Inc and ReCor Medical. The other authors had no conflicts to declare.
