# Supplementary material for: Outcome of Patients With Both Moderate Aortic Stenosis and Moderate Mitral Stenosis
Source: Struct Heart. 2023 Apr 25;7(5):100183. doi: 10.1016/j.shj.2023.100183 (PMC10512010; doi:10.1016/j.shj.2023.100183)
Supplement: Supplemental Tables 1–3 [file mmc1.docx]

**Table S1. Univariate and multivariate analysis determining heart failure symptoms in patients with moderate aortic and mitral stenosis excluding patients with aortic valve area < 1.0cm^2^.**

|  | **Univariate analysis** | | | **Multivariate analysis** | | |
| --- | --- | --- | --- | --- | --- | --- |
|  | **OR** | **95% CI** | **p value** | **OR** | **95% CI** | **p value** |
| Age, years | 0.99 | 0.95–1.03 | 0.53 |  |  |  |
| Male | 0.76 | 0.26–2.11 | 0.60 |  |  |  |
| Hypertension | 3.91 | 0.92–26.9 | 0.065 | 3.01 | 0.60-22.8 | 0.19 |
| Diabetes mellitus | 1.12 | 0.41–3.01 | 0.83 |  |  |  |
| End stage renal disease | 1.05 | 0.31–3.34 | 0.94 |  |  |  |
| Atrial fibrillation | 3.12 | 0.91–11.6 | 0.069 | 4.44 | 0.97-23.6 | 0.054 |
| Coronary artery disease | 1.25 | 0.43–3.55 | 0.68 |  |  |  |
| Systolic BP, mm Hg | 1.00 | 0.98–1.03 | 0.74 |  |  |  |
| Hemoglobin, g/dL | 0.92 | 0.69–1.24 | 0.60 |  |  |  |
| BNP, pg/mL | 1.00 | 1.00–1.00 | 0.76 |  |  |  |
| Inactivity | 0.46 | 0.094–1.72 | 0.26 |  |  |  |
| LV end-diastolic volume, mL | 1.00 | 0.99–1.02 | 0.91 |  |  |  |
| LV ejection fraction, % | 0.94 | 0.88–1.00 | 0.046 | 0.92 | 0.84–0.99 | 0.027 |
| Stroke volume index, mL/m^2^ | 0.96 | 0.92–1.00 | 0.032 | 0.95 | 0.90–1.00 | 0.033 |
| Aortic valve mean PG, mm Hg | 0.99 | 0.91–1.07 | 0.73 |  |  |  |
| Mitral valve mean PG, mm Hg | 1.15 | 0.84–1.58 | 0.39 |  |  |  |
| Left atrial volume index, mL/m^2^ | 1.03 | 1.00–1.06 | 0.072 | 1.02 | 0.98–1.06 | 0.31 |
| Right ventricular systolic pressure, mm Hg | 1.05 | 1.01–1.10 | 0.017 | 1.05 | 1.00–1.11 | 0.043 |

BNP: brain natriuretic peptide, BP: blood pressure, CI: confidence interval, LV: left ventricular, OR: odds ratio, PG: pressure gradient

**Table S2. Univariate and multivariate analysis for predictors of the composite of all-cause mortality, HF hospitalization, and aortic or mitral valve interventions in patients with moderate aortic and mitral stenosis excluding patients with aortic valve area < 1.0cm^2^.**

|  | **Univariate analysis** | | | **Multivariate analysis** | | |
| --- | --- | --- | --- | --- | --- | --- |
|  | **HR** | **95% CI** | **p value** | **HR** | **95% CI** | **p value** |
| Age, years | 0.99 | 0.96–1.01 | 0.35 | 0.99 | 0.96–1.02 | 0.47 |
| Male | 1.24 | 0.63–2.35 | 0.52 | 1.17 | 0.58-2.30 | 0.65 |
| Hypertension | 1.13 | 0.51–3.01 | 0.65 |  |  |  |
| Diabetes mellitus | 0.93 | 0.48–1.74 | 0.82 |  |  |  |
| End stage renal disease | 1.32 | 0.60–2.65 | 0.47 |  |  |  |
| Atrial fibrillation | 1.70 | 0.75–3.47 | 0.19 |  |  |  |
| Coronary artery disease | 1.20 | 0.60–2.28 | 0.60 |  |  |  |
| HF Symptoms | 2.24 | 1.19–4.22 | 0.013 | 2.32 | 1.30-4.14 | 0.0045 |
| Hemoglobin, g/dL | 0.86 | 0.71–1.05 | 0.14 |  |  |  |
| BNP, pg/mL | 1.00 | 1.00–1.00 | 0.81 |  |  |  |
| Inactivity | 1.46 | 0.54–3.35 | 0.43 |  |  |  |
| LV end-diastolic volume, mL | 1.00 | 0.99–1.01 | 0.25 |  |  |  |
| LV ejection fraction, % | 1.02 | 0.98–1.06 | 0.37 |  |  |  |
| Stroke volume index, mL/m^2^ | 1.00 | 0.98–1.01 | 0.98 |  |  |  |
| Aortic valve mean PG, mm Hg | 1.04 | 0.99–1.10 | 0.092 |  |  |  |
| Mitral valve mean PG, mm Hg | 1.01 | 0.79–1.28 | 0.91 |  |  |  |
| Left atrial volume index, mL/m^2^ | 1.01 | 0.99–1.03 | 0.20 |  |  |  |
| Right ventricular systolic pressure, mm Hg | 1.01 | 0.99–1.03 | 0.29 |  |  |  |

BNP: brain natriuretic peptide, BP: blood pressure, CI: confidence interval, HF: heart failure, HR: hazard ratio, LV: left ventricular, PG: pressure gradient

**Table S3. Univariate and multivariate analysis for predictors of all-cause mortality in patients with moderate aortic and mitral stenosis excluding patients with aortic valve area < 1.0cm^2^.**

|  | **Univariate analysis** | | | **Multivariate analysis** | | |
| --- | --- | --- | --- | --- | --- | --- |
|  | **HR** | **95% CI** | **p value** | **HR** | **95% CI** | **p value** |
| Age, years | 1.00 | 0.95–1.05 | 0.97 | 1.03 | 0.97–1.09 | 0.35 |
| Male | 2.01 | 0.66–5.87 | 0.21 | 2.08 | 0.60-6.92 | 0.24 |
| Hypertension | 0.60 | 0.18–2.71 | 0.47 |  |  |  |
| Diabetes mellitus | 1.07 | 0.35–3.11 | 0.90 |  |  |  |
| End stage renal disease | 1.14 | 0.25–3.89 | 0.85 |  |  |  |
| Atrial fibrillation | 1.18 | 0.18–4.51 | 0.83 |  |  |  |
| Coronary artery disease | 1.02 | 0.28–3.06 | 0.98 |  |  |  |
| HF Symptoms | 0.73 | 0.20–2.18 | 0.58 |  |  |  |
| Hemoglobin, g/dL | 0.68 | 0.48–0.97 | 0.035 | 0.69 | 0.47-0.98 | 0.038 |
| BNP, pg/mL | 1.00 | 1.00–1.00 | 0.44 |  |  |  |
| Inactivity | 2.45 | 0.67–7.42 | 0.16 |  |  |  |
| LV end-diastolic volume, mL | 1.01 | 0.99–1.02 | 0.32 |  |  |  |
| LV ejection fraction, % | 1.03 | 0.97–1.10 | 0.29 |  |  |  |
| Stroke volume index, mL/m^2^ | 1.02 | 0.99–1.03 | 0.13 |  |  |  |
| Aortic valve mean PG, mm Hg | 1.04 | 0.95–1.13 | 0.36 |  |  |  |
| Mitral valve mean PG, mm Hg | 1.00 | 0.61–1.51 | 0.99 |  |  |  |
| Left atrial volume index, mL/m^2^ | 1.00 | 0.97–1.03 | 0.82 |  |  |  |
| Right ventricular systolic pressure, mm Hg | 1.00 | 0.96–1.04 | 0.99 |  |  |  |

BNP: brain natriuretic peptide, BP: blood pressure, CI: confidence interval, HF: heart failure, HR: hazard ratio, LV: left ventricular, PG: pressure gradient
